# TNF-α antagonist attenuates systemic lipopolysaccharide-induced brain white matter injury in neonatal rats

**DOI:** 10.1186/s12868-019-0529-1

**Published:** 2019-08-30

**Authors:** Seung Han Shin, Ee-Kyung Kim, Kyung-yup Lee, Han-Suk Kim

**Affiliations:** Department of Pediatrics, Seoul National University College of Medicine, Seoul National University Children’s Hospital, 101 Daehak-ro, Jongno-gu, Seoul, 110-769 South Korea

**Keywords:** Newborn, White matter injury, Systemic inflammation, TNF-α antagonist

## Abstract

**Background:**

Systemic inflammation is an important risk factor for neurodevelopmental impairments in preterm infants. Premyelinating oligodendrocytes are main building blocks of white matter in preterm infants and vulnerable to oxidative stress and excitotoxic stress. Tumour necrosis factor-α (TNF-α) plays important roles in systemic inflammation and local inflammation leading to apoptosis of premyelinating oligodendrocytes and white matter injury (WMI) in brain tissue. This study was conducted to investigate whether etanercept, a TNF-α antagonist, could attenuate systemic lipopolysaccharide (LPS)-induced WMI in the immature brain.

**Results:**

We found that intraperitoneal LPS administration caused systemic and local inflammation in brain tissue. Subsequent etanercept treatment significantly attenuated LPS-induced inflammation in brain tissue as well as in systemic circulation. Intraperitoneal LPS also induced microgliosis and astrocytosis in the cingulum and etanercept treatment reduced LPS-induced microgliosis and astrocytosis. Additionally, systemic LPS-induced apoptosis of oligodendrocyte precursor cells was observed, which was lessened by etanercept treatment. The concentration of etanercept in the CSF was higher when it was administrated with LPS than when administrated with a vehicle.

**Conclusions:**

It appears that etanercept reduce WMI in the neonatal rat brain via attenuation of systemic and local inflammation. This study provides preclinical data suggesting etanercept-mediated modulation of inflammation as a promising approach to reduce WMI caused by sepsis or necrotizing enterocolitis in preterm infants.

## Background

During the neonatal period, preterm infants are at risk from several morbidities, such as sepsis and necrotizing enterocolitis, that are associated with neurodevelopment impairments [1, 2], which have been shown to be mediated by white matter injury (WMI) in the premature brain [2, 3]. The pathogenesis of WMI is characterised by a loss of premyelinating oligodendrocytes (pre-OLs), which are particularly vulnerable to oxidative and excitotoxic stress, and activation of glial cells, such as microglia and astrocytes [4, 5].

Several pro-inflammatory cytokines are associated with the activation of glial cells [6]. Post-mortem brain specimens from infants with periventricular WMI exhibited the expression of various cytokines, including tumour necrosis factor-α (TNF-α), during the early stages of white matter disease [7]. The roll of TNF-α in WMI has been reported in several studies. TNF-α mediated the lipopolysaccharide (LPS)-triggered death of pre-OLs in an environment containing major glial cell types [8], and oligodendrocyte progenitors were particularly vulnerable to a combination of TNF-α and interferon-γ by activation of the cell death program [9]. However, most of studies which reported roll of glial cells and TNF-α in the death of oligodendrocytes during the early developmental phase used in vitro methods with direct stimulation with LPS [8, 10, 11]. Etanercept is a TNF-α antagonist composed of the dimeric fusion protein of the extracellular ligand-binding portion of the soluble 75-kDa TNF-α receptor II and the fragment-crystallisable (Fc) portion of human immunoglobulin (IgG) [12]. Recently, etanercept has been shown to attenuate neural apoptosis, astrocytic and microglial activation, and local inflammation of the brain in several animal studies using traumatic brain injury or ischaemic injury models [13, 14]. In a study of a traumatic brain injury model, injection of intraperitoneal etanercept of 5 mg/kg reduced neuronal degeneration after 1 h, and in a study involving a rat ischemic stroke model, etanercept given intraperitoneally 30 min after insult decreased cerebral infarct [14, 15]. The present study was conducted to establish a neonatal rat model of WMI corresponding to WMI in human preterm infants by inducing systemic inflammation. Furthermore, we hypothesized that administration of TNF-α antagonist following LPS-induced systemic inflammation could attenuate apoptosis of pre-OLs in the inflamed immature brain of newborn rats with subsequent preservation of myelination in the cingulum.

## Results

**Fig. 1:**
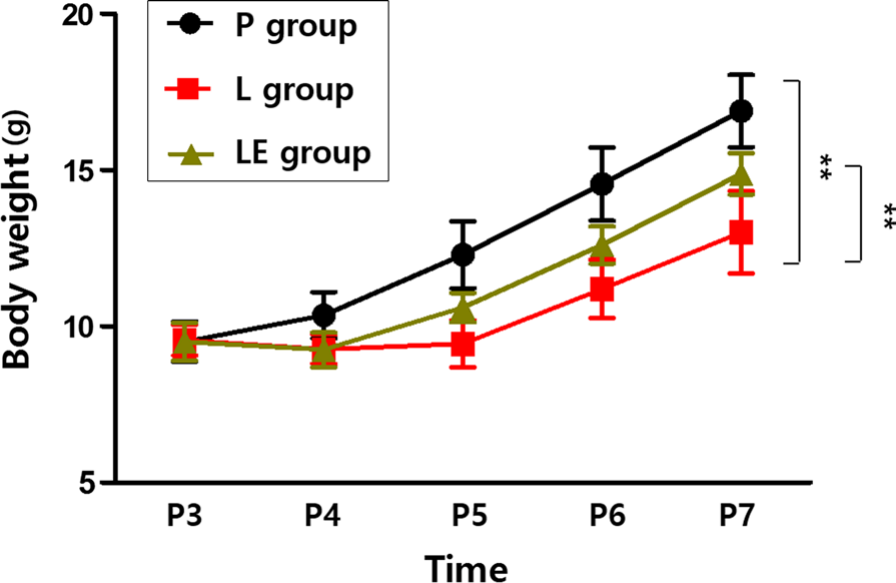
Etanercept-attenuated poor body weight gain in LPS-treated rats. **Represents a significant difference between groups (P < 0.01)

All rats survived until experimental sacrifice. Compared with the P group, rats in both the L and LE groups experienced a slight decrease in body weight on P4 (Fig. 1). Despite the subsequent increase, the body weight of the L group was significantly lower than that of the P group from P5 to P7, and rats in the LE group showed no significant differences in weight during P5 and P7 compared with the P group.

### LPS-induced inflammatory responses were attenuated by etanercept

**Fig. 2:**
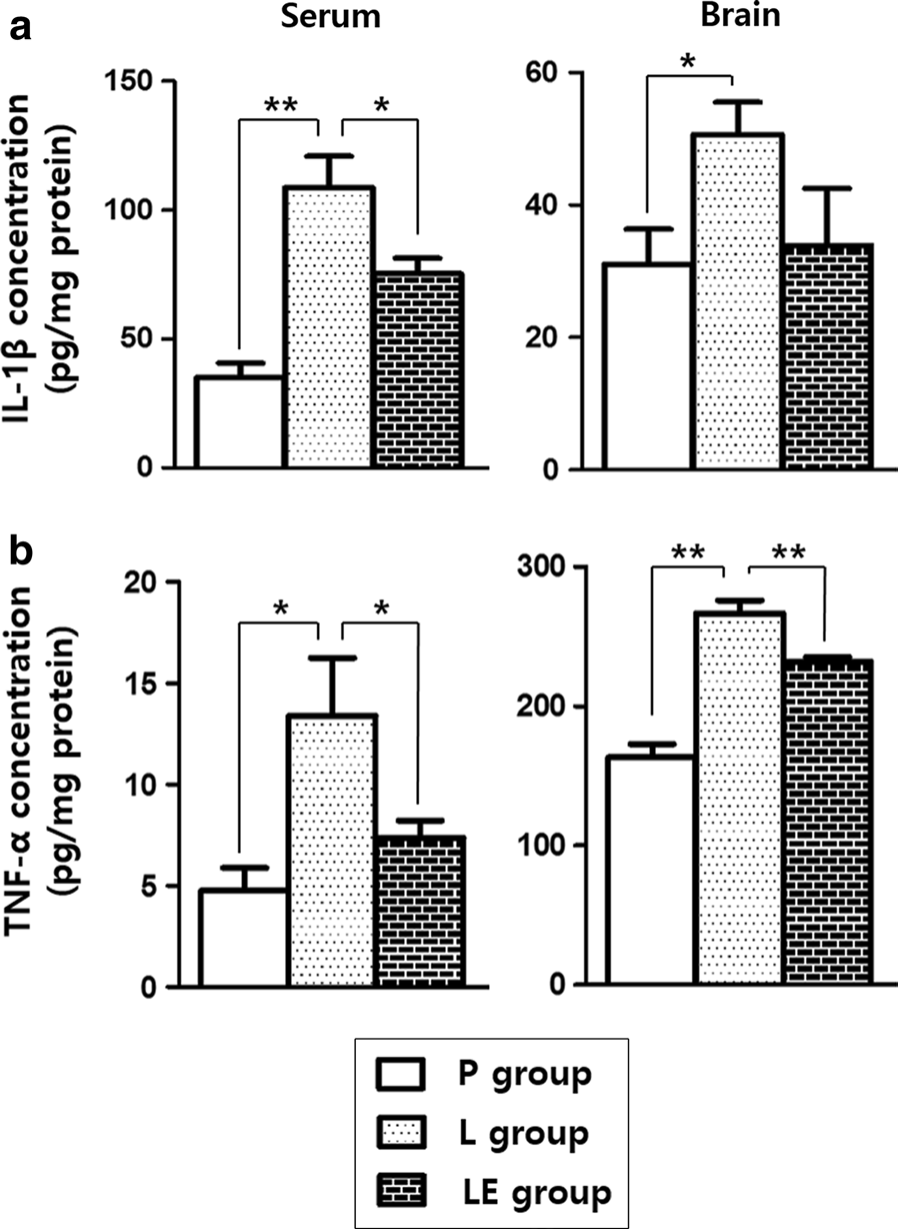
The LPS-induced changes in the levels of inflammatory cytokines in circulation and the brain tissue of rats on P4 (**a** IL-1ß and **b** TNF-α). ** and *represent significant differences between groups (P < 0.01 and P < 0.05, respectively). ANOVA with a Bonferroni test was conducted. There were six rats per group

Systemic exposure to LPS resulted in inflammatory responses in the brain and serum on P4 (Fig. 2a, IL-1ß and Fig. 2b, TNF-α). Serum IL-1ß and TNF-α concentrations were increased in LPS-exposed rats compared with the placebo rats, and the levels in the brain tissue were also significantly increased in LPS-exposed rats. The LE group showed lower IL-1ß levels in serum and lower TNF-α levels in serum and brain tissue than the L group, but attenuation of IL-1ß in the brain was not significant in the LE group.

### Etanercept preserved premyelinating oligodendrocytes during LPS exposure

**Fig. 3:**
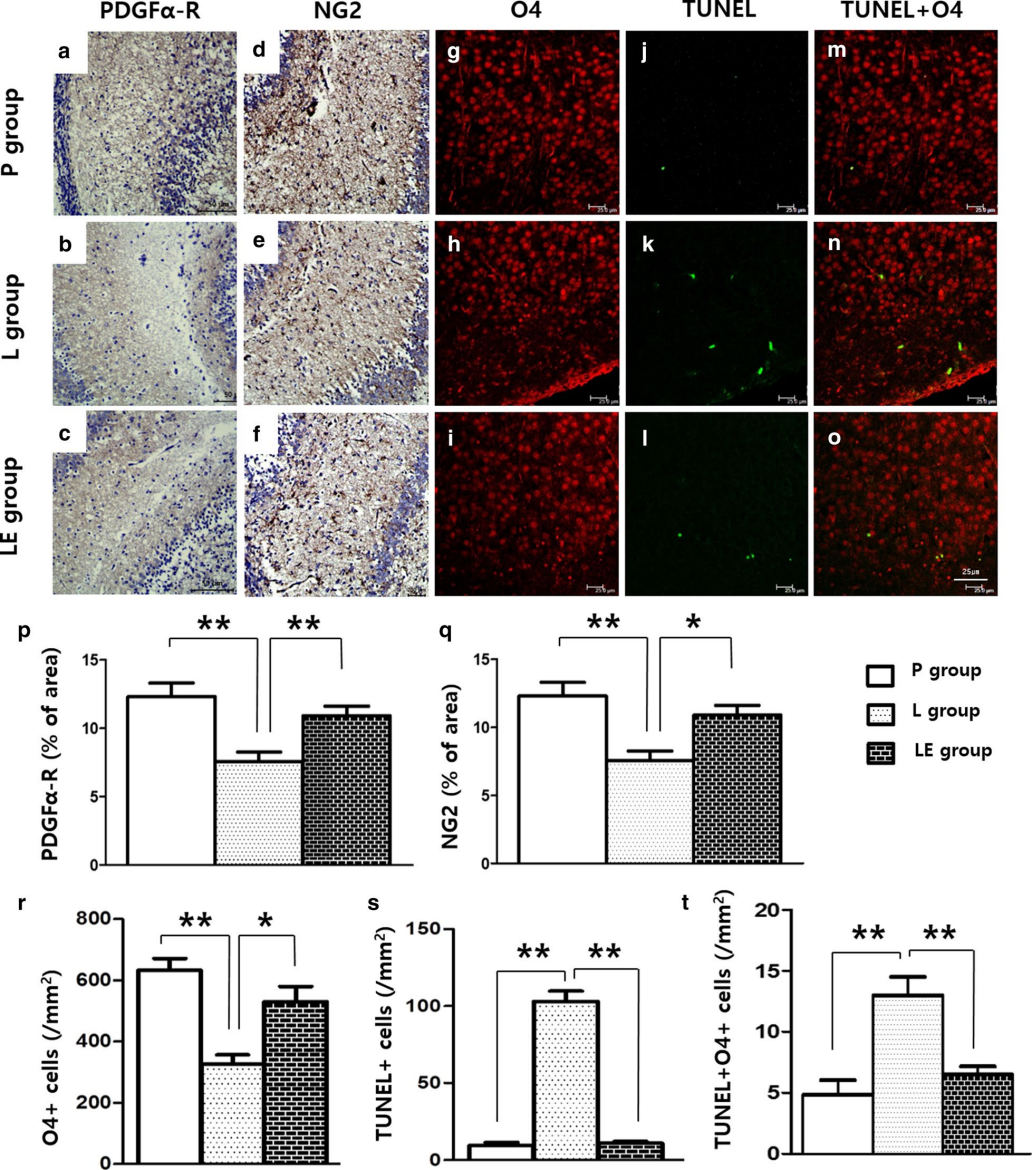
Representative photomicrographs of pre-OLs **a**–**f** by immunohistochemistry using PDGFα-R and NG2 and O4+ oligodendrocyte (**g**–**i**) and TUNEL staining cells **j**–**l** by immunofluorescence (IF) staining of the cingulum (Bregma − 1.0) on P4. Pre-OLs were attenuated in the L group and preserved in the LE group (**p**, **q**). Double-labelling showed that many TUNEL-positive cells were also positive for O4 (**m**–**o**). O4+ oligodendrocytes were decreased in the L group with increased TUNEL-positive cells in the IF staining, and O4+ oligodendrocytes were recovered in the LE group (**r**–**t**). ** and *represent significant differences between groups (P < 0.01 and P < 0.05, respectively). ANOVA with a Bonferroni test was conducted. There were six rats per group

**Fig. 4:**
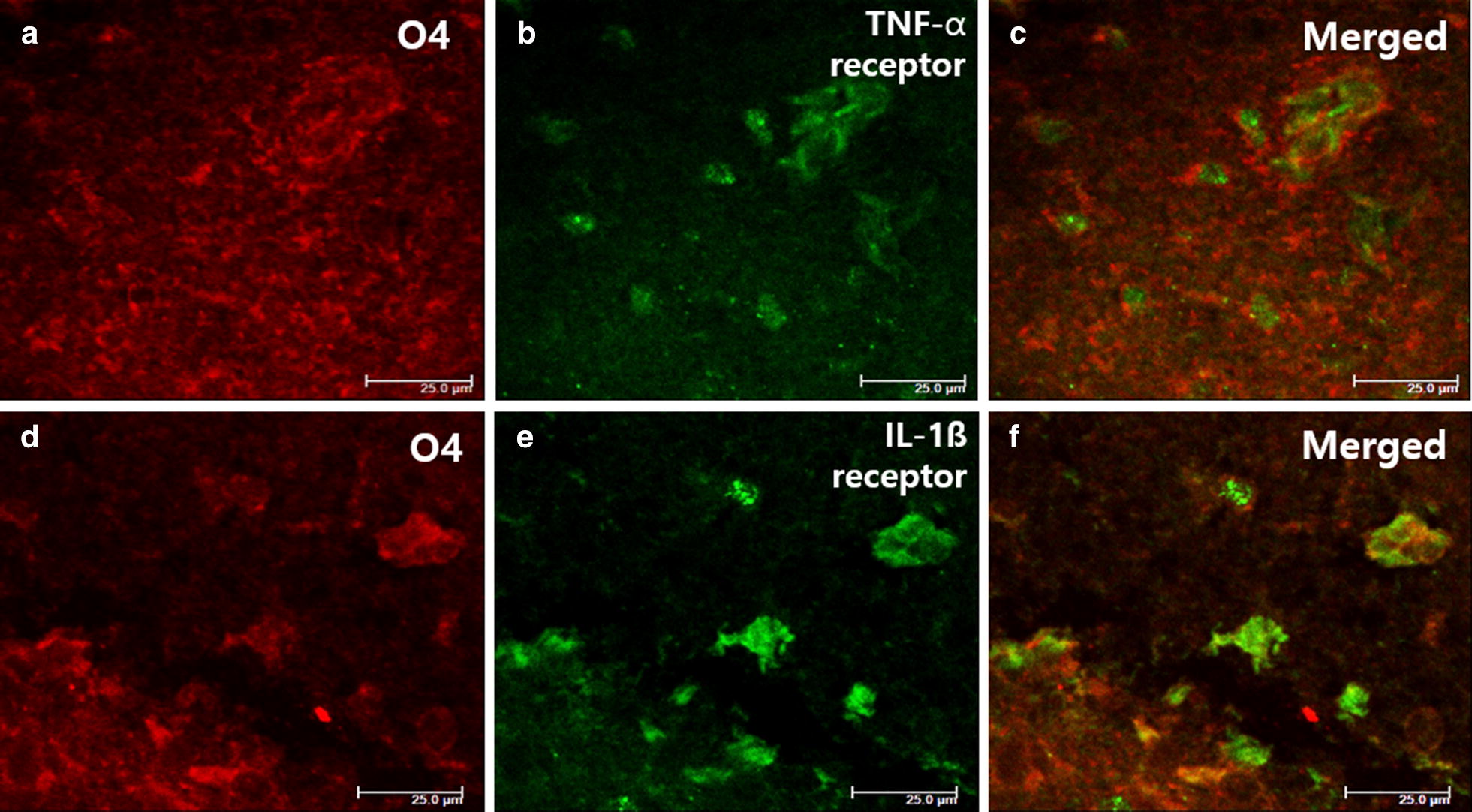
Representative photomicrographs of O4+ oligodendrocyte (**a**, **d**), TNF-α receptor (**b**) and IL-1ß receptor (**e**) immunofluorescence staining of the cingulum (Bregma − 1.0) on P4. Double-labelling showed that many O4+ oligodendrocyte also were positive for TNF-α receptors and IL-1ß receptors (**c**, **f**)

LPS exposure resulted in decreased PDGFα-R and NG2 immunostaining in the brain tissue on P4 (Fig. 3a, b, d, e, p, q), but these were significantly increased in the LE group compared to the L group (Fig. 3c, f, p, q). Decreased distribution of pre-OLs in the LPS-exposed rat brains was also evidenced by decreased O4 immunofluorescence staining, and the O4+ cell was increased in the LE group compared to the L group (Fig. 3g–i, r). TUNEL immunofluorescence staining also was increased in the L group on P4, and this increase was attenuated in the LE group (Fig. 3j–l, s). Double-labelling showed that many TUNEL-positive cells were positive for O4 (Fig. 3n), and the cells that were doubly stained by O4 and TUNEL were decreased in the LE group (Fig. 3m–o, t). Double immunofluorescence staining of TNF-α receptor and IL-1ß receptor with O4 in the L group showed that each receptor was well expressed on the O4+ cells (Fig. 4).

### Etanercept attenuated LPS-induced microgliosis and astrocytosis resulting in improved myelination

**Fig. 5:**
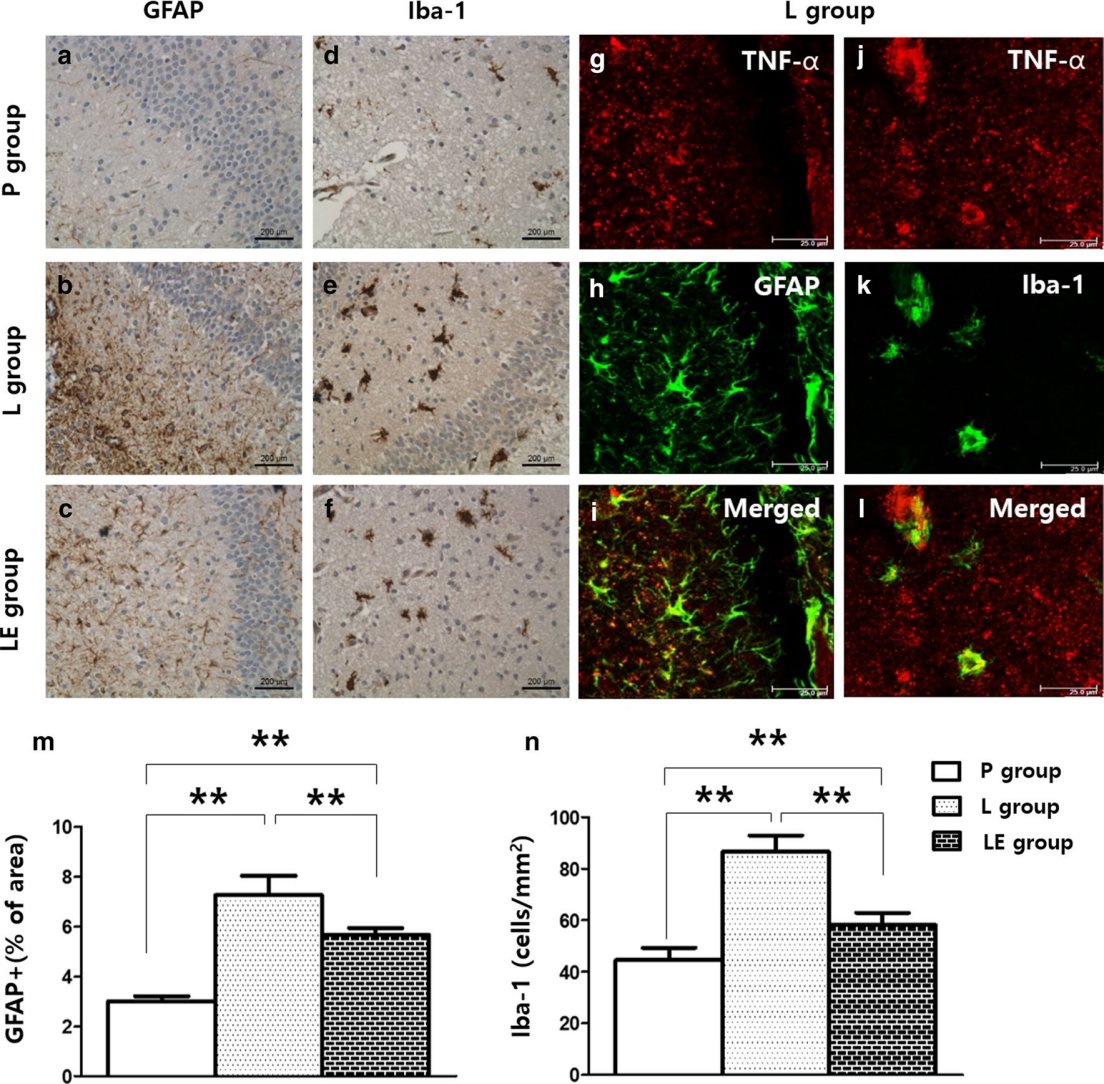
Representative photomicrographs of astrocytosis (**a**–**c**) and microgliosis (**d**–**f**) by immunohistochemistry and TNF-α (**g**, **j**), GFAP (**h**) and Iba-1 (**k**) by immunofluorescence staining of the cingulum (Bregma − 1.0) on P4. Significant increases in the number of reactive astrocytes and microgliosis were observed in the cingulum white matter following neonatal LPS exposure, which was attenuated in the LE group (**m**, **n**). In the L group, GFAP or Iba-1 positive cells were also positive for TNF-α (**i**, **l**). **Represents significant differences between groups (P < 0.01). ANOVA with a Bonferroni test was conducted. There were six rats per group

**Fig. 6:**
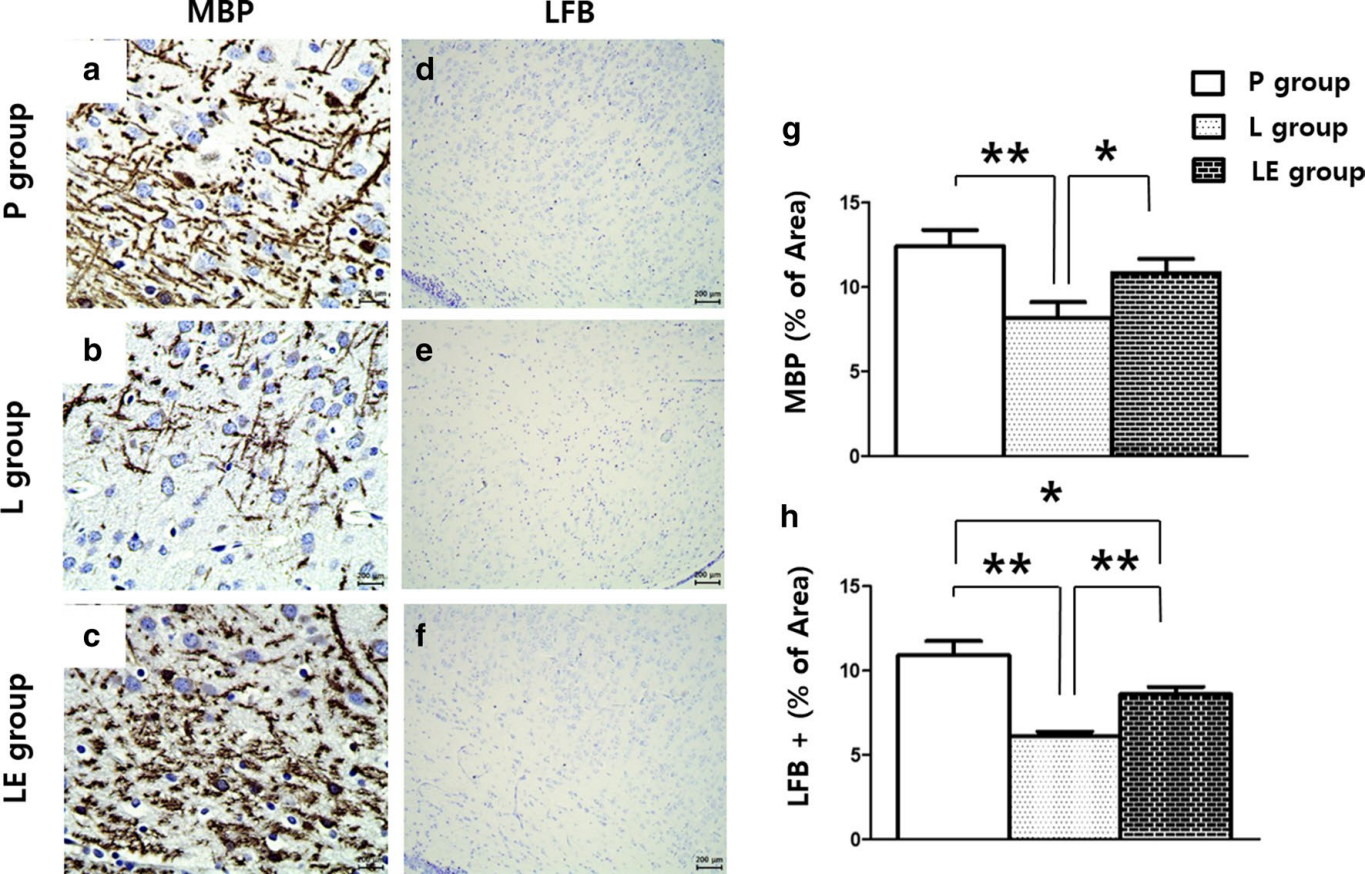
Representative photomicrographs of myelination by immunohistochemistry (**a**–**f**). Cells with MBP and LFB staining were attenuated in the L group and preserved in the LE group (**g**, **h**) of the cingulum (Bregma − 1.0) on P14. ** and *represent significant differences between groups (P < 0.01 and P < 0.05, respectively). ANOVA with a Bonferroni test was conducted. There were six rats per group

A significant increase in the number of reactive astrocytes showing hypertrophy of cellular processes was found in the LPS-exposed rat brains on P4, and this increase was attenuated by etanercept treatment (Fig. 5a–c, m). Iba-1 immunostaining was also increased in the L group, and this increase was attenuated in the LE group (Fig. 5d–f, n). Astrocytosis and microgliosis were still more prominent in the LE group than the control group on P4. Double immunofluorescence labelling in the L group showed that GFAP or Iba-1 positive cells were expressed with TNF-α (Fig. 5g–l). Immunohistochemistry using MBP and LFB on P14 showed that LPS exposure resulted in decreased MBP and LFB and these were significantly increased in the LE group compared to the L group (Fig. 6).

### Etanercept levels in the central nervous system

**Fig. 7:**
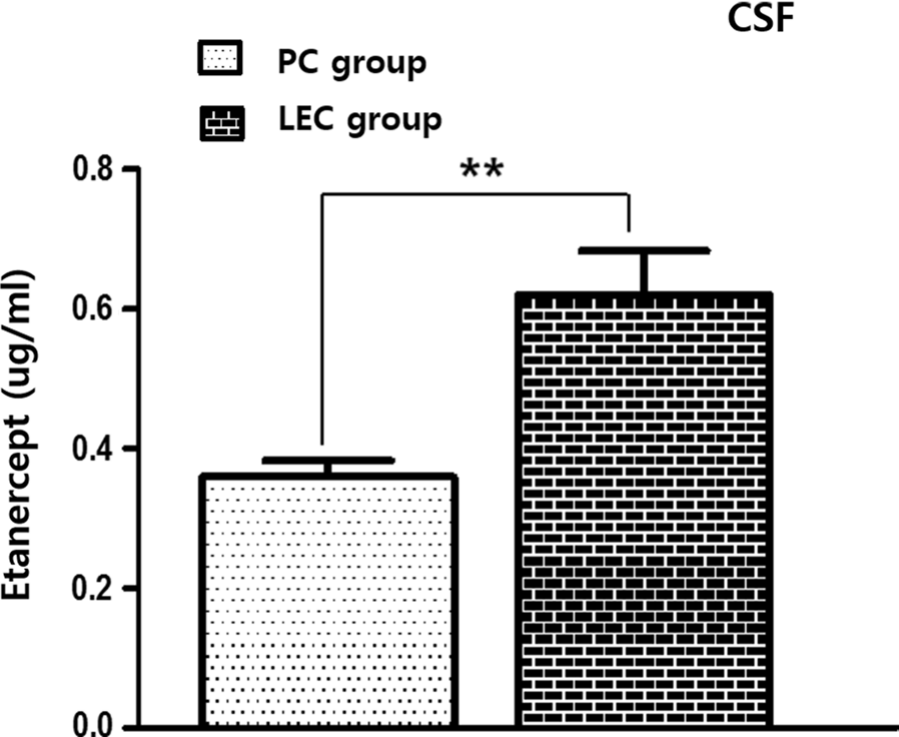
ELISA of etanercept levels in the CSF. The concentration of etanercept in CSF was higher in the LEC group than in the PC group (n = 10 for each group). A t-test was conducted, and **represents a significant difference between groups (P < 0.01)

CSF obtained on P4 was analysed by ELISA to detect etanercept, and the concentration was higher in the LEC group than in the PC group (Fig. 7).

## Discussion

A postnatal newborn rat model of systemic inflammation was reproduced from the study by Fan et al., in which LPS was administered to rats intraperitoneally on day 5 of life [16]. The vulnerability of white matter during systemic inflammation has been related to the presence of pre-OLs at the gestational age of 24–32 weeks in humans, which is equivalent to the same pre-OL phenotype in a newborn rat [17]. Previous studies have suggested that white matter vulnerability at postnatal day 4 in a rat or mouse would correspond to that in preterm infants [18, 19]. In this study, the WMI model was successfully reproduced by IP injection of LPS on P3, and the TNF-α antagonist etanercept attenuated the LPS-induced inflammatory processes with subsequent improvement in myelination.

There was significant systemic inflammation after LPS administration in the newborn rats, which subsequently exhibited poor weight gain during the 1st week of life. Systemic inflammation not only induced local inflammation of the brain but subsequent injury as well, as indicated by increased TUNEL-positive cells in the cingulum white matter. Apoptosis occurred in the O4-positive pre-OLs with decreased myelination in the white matter and was accompanied by microgliosis and astrocytosis in the cingulum, which was consistent with the study by Fan et al. [16]. More immature form of pre-OLs containing NG2 and PDGFα-R also were influenced by systemic LPS and were preserved by TNFα inhibition in this study.

TNF-α was shown to play a critical role in the pathogenesis of WMI because TNF-α signalling is required in LPS-triggered pre-OL death [8]. Pre-OLs do not undergo apoptosis when major glial cell types are absent, which emphasises the importance of intercellular communication in the mechanism of inflammatory pre-OL death. Through this communication, activation of microglia and astrocytes has detrimental effects on neural precursor cells via released TNF-α [20, 21]. The pathogenesis of local brain inflammation during systemic inflammation has not yet been well elucidated, but both direct and indirect pathways of triggering local inflammation have been suggested. Circumventricular organs in the brain might play important roles in the mediation of systemic inflammation and the expression of TNF-α in the brain with CD14 expression [22]. On the other hand, circulating TNF-α has the ability to directly activate the endothelium of the brain’s blood vessels, producing soluble molecules such as prostaglandins with subsequent local inflammation [23]. Many studies used the in vitro methods with direct stimulation with LPS to demonstrate the effect of glial cells and TNF-α on the death of oligodendrocytes during the early developmental phase [8, 10, 11]. In the present study, systemic inflammation by LPS in vivo successfully induced apoptosis of Pre-OLs with the expression of receptors for TNF-α and IL-1β on the Pre-OLs. These changes were accompanied by increased glial cells such as microglia and astrocytes in the brain with the expression of TNF-α (Fig. 5).

Etanercept is a TNF-α antagonist that has already been developed and approved for the treatment of rheumatoid arthritis and several rheumatic diseases, and treatment with etanercept suppressed systemic inflammation and local inflammation in the brains of LPS-treated rats. It decreased microgliosis and astrocytosis and preserved myelination in white matter, subsequently decreasing apoptosis of O4-positive pre-OLs in the LPS- and etanercept-treated newborn rats compared to the LPS-exposed rats that did not receive etanercept. It is unclear whether the effect of etanercept was via suppression of systemic inflammation or direct suppression of local inflammation in the brain or by both mechanisms. However, brain TNF-α levels were strongly suppressed in the LE group compared to the L group, suggesting a role for etanercept in modulating local inflammation. Interestingly, etanercept was more detectable with systemic inflammation (LEC group) than without (PC group). Etanercept is such a large molecule that it is usually unable to penetrate the blood–brain barrier (BBB) [24], but the several studies have demonstrated that the BBB could be disrupted by LPS exposure [25, 26]. This functional change in BBB during systemic inflammation might widen the therapeutic range in the WMI induced by systemic inflammation in preterm infants.

## Conclusions

Although preterm infants are at higher risk of conditions such as sepsis, meningitis and necrotizing enterocolitis during the neonatal period [27], an evidence-based strategy for reducing WMI has not yet been established in this population. Clinical studies have accumulated evidence of an association between serious postnatal systemic inflammation and WMI or neurodevelopmental outcomes in preterm infants [27, 28]. Therefore, controlling undue inflammation in the brain during these episodes might help reduce WMI and subsequent neurodevelopmental impairment in preterm infants. As TNF-α antagonists have already been used to treat a number of neurodegenerative and autoimmune diseases, clinical trials would be worthwhile for this potential treatment for WMI in preterm infants.

## Methods

### Animals

Timed pregnant Sprague–Dawley (SD) rats arrived in the laboratory on day 19 of gestation. Animals were maintained in a room with a 12-h light/dark cycle and a constant temperature (22 ± 2 °C). The day of birth was defined as postnatal day 0 (P0). After birth, the litter size was adjusted to ten pups per litter to minimise its effect on body weight and brain size. All procedures for animal care were conducted in accordance with the National Institutes of Health Guide for the Care and Use of Laboratory Animals and were approved by the Institutional Animal Care and Use Committee at the Seoul National University Hospital. Rats were euthanized by decapitation when the experimental procedure was finished.

### Chemicals

LPS was purchased from Sigma (St. Louis, MO, USA), and enzyme-linked immunosorbent assay (ELISA) kits were used for immunoassay of interleukin-1ß (IL-1ß: MyBioSource, MBS732184) and TNF-α (MyBioSource, MBS032310). Monoclonal mouse antibodies against O4 (MBS832589) and glial fibrillary acidic protein (GFAP, IF03L) were purchased from MyBioSource (San Diego, CA, USA) and Calbiochem (San Diego, CA, USA), respectively. Monoclonal mouse antibody to neural-glial antigen 2 (NG2, AB5320) was purchased from Millipore (Billerica, MA, USA). Monoclonal rabbit antibody to platelet-derived growth factor-α receptor (PDGFα-R, sc-398206) and rabbit antibody to TNF-α (sc-52746) were purchased from Santa Cruz Biotechnology (Santa Cruz, CA, USA). Rabbit antibody to TNF-α receptor I (ab19139) and antibody against myelin basic protein (MBP, ab-40390) were purchased from abcam (Cambridge, UK). Antibody to IL-1ß receptor (NBP1-32681) was purchased from Novus Biological (Colorado, USA). Antibody against Luxol Fast Blue (LFB, LBC-1-IFU) stain were purchased from ScyTek Laboratories (Logan, UT, USA). Ionised calcium-binding adapter molecule 1 (Iba1, 019-19741) was obtained from FUJIFILM Wako Chemicals USA (Irvine, CA, USA). The terminal deoxynucleotidyl transferase (TdT)-mediated uridine 50-triphosphate-biotin nick end labelling (TUNEL) staining kit was purchased from Millipore (Billerica, MA, USA). An enzyme immunoassay (Enbrel ELISA Q-ETA; Matriks Biotek Laboratories, Ankara, Turkey) was used to quantify the etanercept in cerebral spinal fluid (CSF).

### Animal treatment

An intraperitoneal (IP) injection of 2 mg/kg LPS (from *Escherichia coli*, serotype 055: B5) was administered to SD rat pups of both sexes on day 3 of life (P3) followed by IP injection of etanercept at a dose of 5 mg/kg (LE group) or 100 µl of PBS (L group) [15, 16]. The placebo group (P group) was injected intraperitoneally with 100 µl of 0.9% saline followed by the same volume of PBS (100 µl). The room temperature was maintained at 22 ± 2 °C throughout the experiment. Body weight was measured from P3 to P7, and six rats in each group were selected to measure body weight beyond P4 and were sacrificed on P7. A total of 92 rats from eight litters were used in the present study; one pup from each litter was assigned to each group. Six rats from each group were sacrificed by decapitation on P4 to collect blood and fresh cerebrum without olfactory bulb tissue for ELISA assay [16] as well as immunohistochemistry and immunofluorescence staining. Six rats from each group were sacrificed on P14 for the evaluation of changes in myelination by immunohistochemistry. A satellite experiment was performed to investigate the presence of etanercept in the CSF. In one group (LEC group, n = 10), LPS and etanercept were given intraperitoneally on P3 as above, and in the other group (PC group, n = 10), 100 µl of 0.9% saline and etanercept were given on P3. On P4, under light anaesthesia with zoletil (2 mg/kg) and isoflurane (1.5%), rat pups were placed in a stereotaxic apparatus with a neonatal rat adapter, which has light ear pads (David Kopf, CA, USA). A small scalp incision was made on the skull surface, and the lambda was exposed and micropipetted to obtain 1.5 mm CSF. Then, the CSF was centrifuged at 3000 rpm for 10 s, and the supernatant was collected for analysis.

### Determination of IL-1ß and TNF-α protein levels in brain tissue and blood by ELISA

IL-1ß and TNF-α expression levels were determined by ELISA. Cerebrum without olfactory bulb tissues and blood from each pup were collected on P4. Samples were homogenised by sonication in 1 ml of ice-cold PBS (pH 7.2) and centrifuged at 12,000×*g* for 20 min at 4 °C. The supernatant was collected, and the protein concentration was determined using the BCA method. ELISA was performed following the manufacturer’s instructions, and data were acquired using a 96-well plate reader (VersaMax, California, USA). The cytokine contents are expressed as pg cytokine/mg protein.

### Immunohistochemistry and immunofluorescence

Six rats from each group were sacrificed by transcardiac perfusion. Immunohistochemical analysis was performed on 10-μm tissue sections prepared using a microtome, and deparaffinisation and antigen retrieval were conducted. For immunohistochemistry staining, primary antibodies were used in an antibody dilution buffer in the following dilutions: O4, 1:2000; Iba1, 1:2000; MBP, 1:2000; PDGFα-R, 1:50; NG2, 1:100 and GFAP, 1:1000. The sections were incubated overnight at 4 °C with the primary antibodies and then for 2 h with the REAL-HRP system (DaKo REAL TM EnVision TM Detection System, CA, USA, Peroxidase/DAB+, Rabbit/Mouse K5007). Slides were counterstained with Dako Mayer’s Hematoxylin Histological Staining Reagent. Photomicrographs were captured using a Leica DFC 290 microscope with a digital camera system. Immunofluorescence staining was conducted using TUNEL assay kits. Next, the sections were incubated overnight with anti-O4 antibodies at 4 °C and then incubated with a secondary antibody conjugated to a fluorescent probe (Alexa Fluor 568, 1:200 anti-mouse IgM, Invitrogen, Grand Island, NY, USA) for 2 h in the dark at room temperature. Sections were washed with PBS and then mounted using VECTASHIELD mounting medium (Vector Laboratories, Inc., Burlingame, CA) for visualisation under a fluorescence microscope (Leica TCS SP8). The TUNEL-positive cells appeared green, and the O4-positive cells appeared red. Immunofluorescence staining of GFAP (1:200), Iba-1 (1:200), TNF-α (1:100), TNF-α receptor (1:100), and IL-1ß receptor (1:100) were also conducted as descripted above. Secondary antibodies were conjugated to a fluorescent probe (Alexa Fluor 488; anti-mouse IgG, 488; anti-rabbit IgG, 555; anti-mouse IgG, 594; anti-rabbit IgG, 1:200 Invitrogen, Grand Island, NY, USA). Antigen retrieval and the composition of the antibody dilution buffer were conducted according to the manufacturer’s instructions. Staining intensities were measured in brain cingulum (Bregma − 1.00) using Image J software [29].

### Statistical analysis

SPSS version 22.0 (SPSS for Windows Inc., Chicago, IL, USA) was used for the statistical analysis. Kolmogorov–Smirnov test was used to evaluate the equality of distributions and comparison between two groups was performed by t-test. ANOVAs with Bonferroni comparison tests were used to compare the groups, and the RT-PCR data were analyzed by the 2-delta delta C_T_ method. The results are expressed as the mean ± standard deviation.

## Data Availability

The datasets used and/or analyzed during the current study available from the corresponding author on reasonable request.

## Abbreviations

CSF: cerebral spinal fluid
ELISA: enzyme-linked immunosorbent assay
GFAP: glial fibrillary acidic protein
Iba-1: ionised calcium-binding adapter molecule 1
IP: intraperitoneal
LFB: luxol fast blue
LPS: lipopolysaccharide
NG2: neural-glial antigen 2
PDGFα-R: platelet-derived growth factor-α receptor
Pre-OLs: premyelinating oligodendrocytes
TNF-α: tumor necrosis factor-α
TUNEL: terminal deoxynucleotidyl transferase-mediated uridine 50-triphosphate-biotin nick end labelling
WMI: white matter injury
